# Prevalence and Characterization of *Cryptosporidium* Species and Genotypes in Four Farmed Deer Species in the Northeast of China

**DOI:** 10.3389/fvets.2020.00430

**Published:** 2020-08-10

**Authors:** Wei Zhao, Jie Xu, Mengran Xiao, Jianping Cao, Yanyan Jiang, Huicong Huang, Bin Zheng, Yujuan Shen

**Affiliations:** ^1^Chinese Center for Disease Control and Prevention, National Institute of Parasitic Diseases, Shanghai, China; ^2^Chinese Center for Tropical Diseases Research, Shanghai, China; ^3^WHO Collaborating Centre for Tropical Diseases, Shanghai, China; ^4^National Center for International Research on Tropical Diseases, Ministry of Science and Technology, Shanghai, China; ^5^Key Laboratory of Parasite and Vector Biology, Ministry of Health, Shanghai, China; ^6^Department of Parasitology, Wenzhou Medical University, Wenzhou, China

**Keywords:** *Cryptosporidium*, deer, zoonotic, genetic characterization, human

## Abstract

Cryptosporidiosis is a major public health problem in humans and animals. Information on the prevalence and molecular diversity of *Cryptosporidium* in farmed deer in northeastern China is limited. In this study, the prevalence of these parasites was investigated in four farmed deer species, including 125 reindeer, 109 red deer, 86 sika deer, and 18 Siberian roe deer by nested PCR amplification of the partial small subunit of ribosomal RNA (SSU rRNA) gene. *C. ubiquitum* isolates were subtyped using nested PCR and sequence analysis of the 60-kDa glycoprotein (*gp60*) gene. The overall prevalence of *Cryptosporidium* was 7.1%, with 15.1% for sika deer, 4.0% for reindeer, 4.6% for red deer, and 5.6% for roe deer. *C. ubiquitum* (*n* = 4), *C. xiaoi* (*n* = 2), and *Cryptosporidium* deer genotype (*n* = 18) were identified. All four *C. ubiquitum* isolates belonged to the XIIa subtype (*n* = 4). This study confirms that *Cryptosporidium* deer genotype is widely occurring in deer in the investigated areas. Presence of zoonotic *C. ubiquitum* XIIa subtype indicates that farmed deer represent potential source of zoonotic cryptosporidia and might pose a threat to human health.

## Introduction

*Cryptosporidium* is an important zoonotic protozoan parasite with a cosmopolitan distribution (1). The transmission routes of *Cryptosporidium* spp. are thought to result from fecal–oral transmission of oocysts via direct contact with infected humans or animals, or through the ingestion of contaminated water or food (2). However, the contribution of animal reservoirs to human infections remains unclear and requires clarification (3). PCR-based molecular tools for the genetic characterization of *Cryptosporidium* have enhanced our understanding of *Cryptosporidium* epidemiology, providing information on the host distribution of various species/genotypes and transmission routes/sources (4).

The genetic heterogeneity of the SSU rRNA gene has revealed the existence of ≥39 recognized species of *Cryptosporidium* (5, 6). Some of these species have been identified in both humans and animals (particularly farm animals, such as sheep and cattle). Contact with farmed animals is an identified risk factor for human cryptosporidiosis, and many outbreaks have been documented, often involving veterinary students and students at farm schools (7–9). The identification of *Cryptosporidium* species/genotypes in farmed animals has enhanced our understanding of the transmission of *Cryptosporidium*.

Commercially farmed deer species vary according to region, but some species such as red deer (*Cervus elaphus*), sika deer (*Cervus nippon*), and reindeer (*Rangifer tarandus*) are farmed across the globe (http://www.fao.org/docrep/004/X6529E/X6529E02.htm). Currently, *Cryptosporidium* studies in deer have focused on wild or free-ranging species rather than farmed animals (10). Genetic studies of *Cryptosporidium* from deer showed that eight species (*C. parvum, C. hominis, C. ubiquitum, C. muris, C. andersoni, C. occultus, C. bovis*, and *C. ryanae*) and four unnamed *Cryptosporidium* genotypes (deer genotype, muskrat II genotype, *C. hominis*-like genotype, and caribou genotype) are prevalent, suggesting that deer infection with *Cryptosporidium* poses a potential threat to human health (Table 1) (11–25).

**Table 1:** Prevalence and distribution of *Cryptosporidium* species/genotypes in deer according to country.

**Location**	**Deer species**	**No. of positive / No. of examined (%)**	***Cryptosporidium* spp./genotype (n)**	**References**
Australia	Deer	21/1,563 (1.3)	*C. ryanae* (15); *C. ubiquitum* (3); *C. hominis* (2); *C. suis-*like (1)	(11)
Canada	Caribou	3/49 (6.1)	*Cryptosporidium* caribou genotype	(12)
China	Sika deer	2/83 (2.4)	*C. ubiquitum* (1)	(13)
		9/818 (1.1)	*Cryptosporidium* deer genotype (9)	(14)
		41/599 (6.8)	*Cryptosporidium* deer genotype (21); *C. parvum* (11); *C. andersoni* (5); *C. ubiquitum* (2); *C. muris* (1); *C. suis-*like (1)	(15)
	Père David's deer	3/47 (6.4)	*Cryptosporidium* deer genotype (2); *C. ubiquitum* (1)	(15)
		2/128 (1.6)	*C. parvum* (1); *Cryptosporidium* deer genotype (1)	(16)
	Red deer	1/16 (6.3)	*Cryptosporidium* deer genotype (1)	(15)
Czech Republic	Red deer	6/136 (4.4)	*C. ubiquitum* (5); *C. muris* (1)	(17)
	White-tailed deer	3/26 (11.5)	*Cryptosporidium* deer genotype (2); *C. muris* (1)	(17)
Japan	Sika deer	25/319 (7.8)	*Cryptosporidium* deer genotype (18)	(19)
Nepal	Swamp deer	4/32 (12.5)	*C. ubiquitum* (4)	(20)
Spain	Roe deer	9/212 (4.2)	*C. bovis* (3) *C. ryanae* (3)	(21)
UK	Red deer	16/20 (80.6)	*C. parvum* (14); *Cryptosporidium* deer genotype (1); *C. parvum* and *Cryptosporidium* deer genotype (1)	(22)
	Roe deer	2/6 (33.3)	*C. parvum*	(22)
UK	Roe deer	2/46 (7.7)	*C. ubiquitum* (1); *Cryptosporidium* deer genotype (1)	(23)
USA	White-tailed deer	2/91 (2.2)	*C. parvum* (1); *Cryptosporidium* deer genotype and *C. hominis*-like (1)	(24)
		10/80 (12.5)	*Cryptosporidium* deer genotype (10)	(25)
		10/91 (11.0)	*C. ubiquitum* (4); *C. parvum* (1); *Cryptosporidium* Muskrat II genotype (1)	(18)

*C. parvum, C. ubiquitum, and Cryptosporidium muskrat II genotype reported as Genotype 2, Genotype 3, and Genotype 4, respectively, in the publication (18)*.

In China, reindeer, sika deer, red deer, and Siberian roe deer (*Capreolus pygargus*) are commonly farmed in the northeast of China (14). However, reports on *Cryptosporidium* infections in these animals are limited (13–16). This study investigated the prevalence and species/genotypes distribution of *Cryptosporidium* in these four deer species in northeastern China.

## Materials and Methods

### Collection of Fecal Specimens

**Figure 1:**
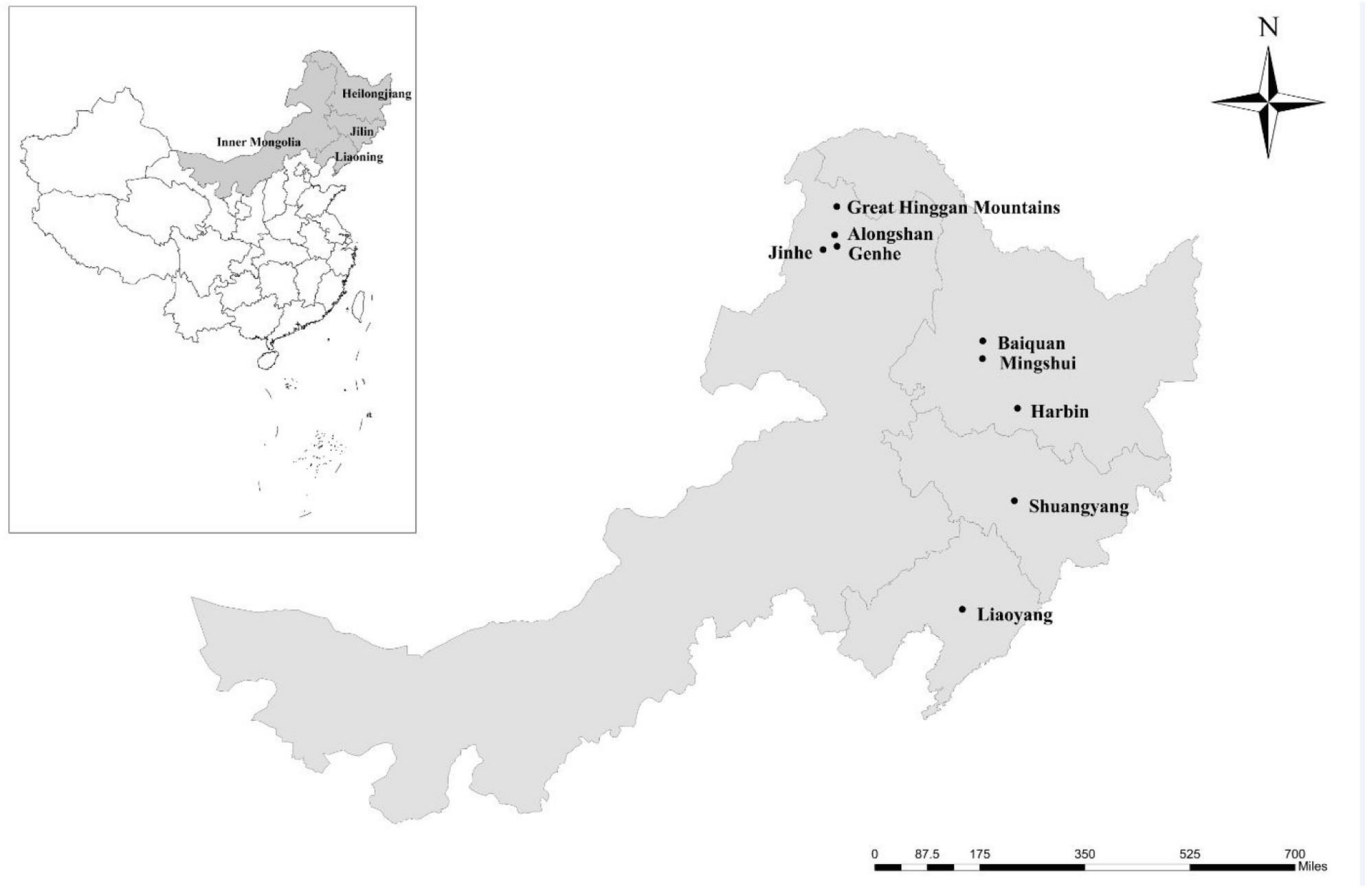
Specific locations where samples were collected in this study. • Sampling points.

From 1 May 2012 to 31 March 2016, 338 fresh fecal specimens (approximately 10 g) were collected from four farmed deer species, including 125 reindeer, 109 red deer, 86 sika deer, and 18 Siberian roe deer from 10 farms located in nine areas of four provinces in the northeast of China (Table 1 and Figure 1). All fecal specimens were collected from the ground immediately after defecation using sterile disposable latex gloves. To avoid the contamination from the ground, only the parts that do not touch the ground were collected. The number of collected specimens accounted for ~30% of the adult or young deer on each farm. All specimens were transported to the laboratory in coolers with ice packs within 24 h and were stored at 4°C (<24 h). The source, age, and health status (with or without diarrhea) of each deer were recorded during sampling. The ages of the adults ranged from 3 to 5 years, and the ages of the young deer ranged from 1 to 6 months (no animals aged 6 months to 3 years were sampled).

### DNA Extraction

Total genomic DNA from each fecal sample (~200 mg) was extracted using a QIAamp DNA Mini Stool Kit (Qiagen, Hilden, Germany) according to the manufacturer's recommendations. Eluted DNA was stored at −20°C prior to PCR analysis. All analysis of fecal DNA extractions was performed in a biosafety level 2 laboratory.

### PCR Amplification of *Cryptosporidium*

*Cryptosporidium* was detected by nested PCR amplification of the SSU rRNA gene fragment of ~830 bp. Primers and cycle parameters were designed by Xiao and colleagues (26). A fragment of ~948 bp of the 60-kDa glycoprotein *(gp60)* gene was used to identify *C. ubiquitum* subtypes via nested PCR amplification using the primers described by Li et al. (27). TaKaRa Taq DNA polymerase (TaKaRa Bio Inc., Tokyo, Japan) was used for all PCRs. PCR amplifications were performed with positive (chicken-derived *C. bailey* DNA) and negative controls (no DNA water). PCR products were visualized on a UV transilluminator following electrophoresis on 1.5% agarose gels stained with GelStrain (Trans Gen Biotech, Beijing, China).

### DNA Sequencing and Analysis

Positive PCR amplicons were transferred to Sangon Biotech Co. Ltd. (Shanghai, China) for sequencing. The accuracy of the sequencing data was confirmed by bi-directional sequencing. Species and genotypes of *Cryptosporidium* were identified through the comparison of the nucleotide sequences deposited at the National Center for Biotechnology Information (NCBI) using the BLAST (http://blast.ncbi.nlm.nih.gov/Blast.cgi).

### Statistical Analysis

Prevalence was calculated according to deer species and age (young *vs*. adult deer). Categorical variables were expressed as numbers of cases (percentages), and frequencies were compared using chi-square tests. Statistical significance was established at a *p* ≤ 0.05. Analyses were performed using SPSS statistical software package version 19.0 (IBM Corporation, Somers, NY, USA).

## Results

### Prevalence of Cryptosporidium

*Cryptosporidium* was detected in all four deer species as assessed by nested PCR amplification of the SSU rRNA gene. In total, *Cryptosporidium* spp. were found in 7.1% (24/338) of deer, with 15.1% (13/86) in sika deer, 4.0% (5/125) in reindeer, 4.6% (5/109) in red deer, and 5.6% (1/18) in roe deer (Table 2). Significant differences in prevalence were observed among species as assessed by chi-square tests (χ^2^ = 12.8, *P* = 0.008). *Cryptosporidium* was identified in three farms for sika deer (7.7–20.1%), two farms for reindeer (1.7 and 7.3%), and two farms for red deer (6.8 and 8.3%).

**Table 2:** Prevalence and species/genotypes of *Cryptosporidium* in the investigated deer species in China.

**Deer species (Latin name)**	**Location**	**Positive/examined (%)**	***Cryptosporidium* spp. /genotype (*n*)**
Red deer (*Cervus elaphus*)	Great Hinggan Mountains	2/44 (6.8)	Deer genotype (2)
	Harbin	0/5	−
	Liaoyang	3/60 (8.3)	Deer genotype (3)
Subtotal		5/109 (4.6)	Deer genotype (5)
Reindeer (*Rangifer tarandus*)	Alongshan	3/41 (7.3)	*C. ubiquitum* (2); Deer genotype (1)
	Genhe	2/59 (3.4)	*C. xiaoi* (2)
	Jinhe	0/25	−
Subtotal		5/125 (4.0)	*C. ubiquitum* (2); Deer genotype (1); *C. xiaoi* (2)
Roe deer (*Capreolus pygargus*)	Liaoyang	1/18 (5.6)	Deer genotype (1)
Sika deer (*Cervus nippon*)	Baiquan	4/31 (12.9)	Deer genotype (2); *C. ubiquitum* (2)
	Mingshui	2/21 (7.7)	Deer genotype (2)
	Shuangyang	7/34 (20.1)	Deer genotype (7)
Subtotal		13/86 (15.1)	Deer genotype (11); *C. ubiquitum* (2)
Total		24/338 (7.1)	Deer genotype (19); *C. ubiquitum* (4); *C. xiaoi* (2)

The overall prevalence of *Cryptosporidium* in young deer (17.1%; 13/76) was significantly higher than that in adults (4.1%; 10/244) (χ^2^ = 14.70, *P* < 0.05). Statistical differences in prevalence were observed in sika deer between the two age groups (41.2 vs. 8.7%) (χ^2^ = 8.83, *P* < 0.05), while no significant differences were observed in reindeer (7.7 vs. 1.2%) (χ^2^ = 1.89, *P* > 0.05) and red deer (15.0 vs. 2.2%) (χ^2^ = 3.50, *P* > 0.05). No samples from young roe deer were used in this study (Table 3). All deer had no diarrhea at the time of sampling.

**Table 3:** Prevalence and *Cryptosporidium* species/genotypes in the four species of deer according to age.

**Group**	**Red deer**		**Reindeers**		**Roe deer**		**Sika deer**	
	**Positive/examined (%)**	**Species/genotypes**	**Positive/examined (%)**	**Species/genotypes**	**Positive/examined (%)**	**Species/genotypes**	**Positive/examined (%)**	**Species/genotypes**
Youths	3/20 (15.0)	Deer genotype (3)	3/39 (7.7)	Deer genotype (1); *C. ubiquitum* (2)	–	–	7/17 (41.2)	Deer genotype (5); *C. ubiquitum* (2)
Adults	2/89 (2.2)	Deer genotype (2)	2/86 (2.3)	*C. xiaoi* (2)	1/18 (5.6)	Deer genotype (1)	6/69 (8.7)	Deer genotype (6)
Total	5/109 (4.6)	Deer genotype (5)	5/125 (4.0)	*C. ubiquitum* (2); *C. xiaoi* (2); Deer genotype (1)	1/18 (5.6)	Deer genotype (1)	13/86 (15.1)	Deer genotype (11); *C. ubiquitum* (2)

### Genotyping and Subtyping of *Cryptosporidium*

All 24 *Cryptosporidium-*positive specimens were successfully sequenced at the SSU rRNA gene. Through sequence analysis, three species/genotypes were identified: *C. ubiquitum* (*n* = 4), *C. xiaoi* (*n* = 2), and *Cryptosporidium* deer genotype (*n* = 18). All 18 sequences of *Cryptosporidium* deer genotype were identical to each other, showing 100% homology with those from a white-tailed deer in the Czech Republic (KR260681), and a sika deer (KX259127), a David's deer (KX259128), and a red deer (KX259129) in China. The four *C. ubiquitum* isolates and two *C. xiaoi* isolates had 100% similarity with those from feral deer in Australia (MG516762) and goats in China (KM199754), respectively. *C. ubiquitum* isolates were further subtyped through amplification of the *gp60* gene. All four *C. ubiquitum* isolates were successfully amplified and sequenced, and all belonged to the XIIa subtype, sharing 100% homology with previous XIIa subtypes derived from Tibetan sheep in China (KU052815).

*Cryptosporidium* deer genotype was the dominant genotype (75.0%, 18/24) and had the widest host and geographical distribution, being detected in four deer species and in 6/10 of the investigated areas (Table 1). *C. ubiquitum* was identified in young sika deer and reindeer (two each), while *C. xiaoi* was found in two adult reindeer only (Table 3).

## Discussion

Epidemiological investigations of *Cryptosporidium* in deer have been documented in over eight countries since the first detection of *Cryptosporidium* in deer in Scotland in 1981 (10, 28). However, the number of studies characterizing *Cryptosporidium* spp. in deer is low (Table 1) (11–25). In this study, the average prevalence of *Cryptosporidium* in the different deer species was 7.1% (15.1% for sika deer, 4.0% for reindeer, 4.6% for red deer, and 5.6% for roe deer), which was higher than previous studies in China (13–16). Meanwhile, the prevalence of *Cryptosporidium* was higher in young animals (17.1%) than adults (3.7%), consistent with previous studies (19, 21, 29). The assessment of *Cryptosporidium* infection in sika deer from Japan showed that fawns had a higher prevalence (16.1%) than yearlings (6.4%) and adults (4.7%) (19). Likewise, studies from Norway demonstrated that 6.2% of roe deer were infected with *Cryptosporidium*, with prevalence in calves significantly higher than in yearlings and adults (29). *Cryptosporidium* infection was more prevalent in juvenile deer compared to roe deer in Spain, although the differences were not significant (21). The prevalence of *Cryptosporidium* in young deer was higher than those of adults, most likely due to the underdeveloped immune systems of the young animals.

In this study, *Cryptosporidium* deer genotype, *C. ubiquitum*, and *C. xiaoi* were identified. *Cryptosporidium* deer genotype had the highest frequency and widest distribution. A number of lines of evidence supported the observation that deer are the only animal host for *Cryptosporidium* deer genotype (15). This genotype has been identified in sika deer from China and Japan, white-tailed deer from the Czech Republic and USA, roe deer from the UK, red deer from China and the UK, and Père David's deer from China (13–16, 19, 22, 23).

*Cryptosporidium ubiquitum*, previously named *Cryptosporidium* cervine genotype, has a broad host range that includes carnivores, rodents, primates, and domestic and wild ruminants (13, 27). To date, *C. ubiquitum* has been detected in sika deer in China, white-tailed deer in the USA, swamp deer in Nepal, roe deer in the UK, red deer in the Czech Republic, and deer (sambar deer, red deer, and fallow deer) in Australia (11, 13, 17, 18, 20). Human infections have been documented in the UK, Slovenia, the USA, Canada, Spain, and New Zealand (13, 27). *Cryptosporidium ubiquitum* is also one of the most common *Cryptosporidium* species in drinking water in China (30). In this study, four *C. ubiquitum* isolates were identified as zoonotic XIIa subtype, which were identified in domestic and wild ruminants, rodents, humans, and water samples (5, 30–32). The facts above indicate that the deer infected with this subtype represent infection reservoir and might potentially pose a threat to human health.

*Cryptosporidium xiaoi* (previously named as *C. bovis*-like or *C. bovis*) from sheep was initially identified by Chalmers and colleagues in 2002 and was formally described as a species in 2009, which is genetically distinct but closely related to *C. bovis* (33, 34). *Cryptosporidium xiaoi* primarily infected sheep and appeared asymptomatic, but has also been reported in yaks, goats, fish, and kangaroos (35–38). Up to now, only two cases of cryptosporiosis caused by *C. xiaoi* have been reported in HIV/AIDS patients from Ethiopia (39). *Cryptosporidium xiaoi* was detected as a dominant species in small ruminants in some other African countries including Egypt and Tanzania, in addition to Asian countries including Bangladesh and China (3, 5, 40, 41). Similarly, *C. xiaoi* was the major *Cryptosporidium* species in small ruminants in Europe countries including France, Greece, Poland and Norway, and Australia (40, 42–44). Our findings of *C. xiaoi* in reindeer indicate that this species might have a more extensive host spectrum than previously expected. The source of *C. xiaoi* infection and its transmission dynamics require further investigation to elucidate the cross-species transmission potential of *C. xiaoi* in deer and other animals, including humans in China.

## Conclusions

This study demonstrates the prevalence and species/genotypes distribution of *Cryptosporidium* in four deer species in the northeast of China. Two species (*C. ubiquitum* and *C. xiaoi*) and one genotype (*Cryptosporidium* deer genotype) of *Cryptosporidium* were identified. Additionally, the zoonotic subtype of *C. ubiquitum* XIIa subtype was found in the reindeer and sika deer. Presence of the zoonotic subtype *C. ubiquitum* XIIa in reindeer and sika deer suggests the importance of deer as a potential source of zoonotic cryptosporidia in the environment.

## Data Availability Statement

All 24 sequences in the article are 100% homology with some sequences in the GenBank database. These data were included in the article.

## Ethics Statement

The study protocol was approved by the Laboratory Animal Welfare and Ethics Committee (LAWEC), National Institute of Parasitic Diseases, Chinese Center for Disease Control and Prevention, China (reference no. 2012-12). Prior to initiating the study, permission was obtained from farm owners or managers. No deer were harmed during specimen collection.

## Author Contributions

BZ and YS conceived, designed the experiments, and revised the manuscript. WZ performed the experiments and wrote the paper. WZ, JX, MX, and YJ analyzed the data. JC and HH contributed reagents and material analysis tools. All authors read and approved the final version of the manuscript.

## Conflict of Interest

The authors declare that the research was conducted in the absence of any commercial or financial relationships that could be construed as a potential conflict of interest.
